# DSC-DeepLabv3+: a lightweight semantic segmentation model for weed identification in maize fields

**DOI:** 10.3389/fpls.2025.1647736

**Published:** 2025-09-03

**Authors:** Haitao Fu, Xiaoyao Li, Li Zhu, Xin Pan, Tuo Wu, Wen Li, Yuxuan Feng

**Affiliations:** ^1^ College of Information Technology, Jilin Agricultural University, Changchun, China; ^2^ BaichengAgricultural and Rural Information Center, Baicheng, China

**Keywords:** weed recognition, lightweight semantic segmentation, DeepLabV3+, feature fusion, attention mechanisms

## Abstract

**Introduction:**

Weeds compete with crops for water, nutrients, and light, negatively impacting maize yield and quality. To enhance weed identification accuracy and meet the requirements of precision agriculture, we propose a lightweight semantic segmentation model named DSC-DeepLabv3+.

**Methods:**

MobileNetV2 is adopted as the backbone, and standard convolutions in atrous spatial pyramid pooling (ASPP) and decoder modules are replaced with depthwise separable dilated convolutions (DSDConv), significantly reducing model complexity and improving segmentation efficiency. To capture rich contextual information, strip pooling is incorporated into the ASPP module, forming the strip pooling–atrous spatial pyramid pooling (S-ASPP) structure. In addition, a convolutional block attention module (CBAM) is introduced to refine feature representations, and multi-scale features are further fused using the CBAM–Cascade Feature Fusion (C-CFF) module to improve semantic understanding.

**Results:**

Experimental results show that the proposed model reduces the number of parameters from 54.714M to 2.89M and decreases the computational cost from 167.139 GFLOPs to 15.326 GFLOPs, while achieving an inference speed of 42.89 FPS and a mean Intersection over Union (mIoU) of 85.57%.

**Discussion:**

These results demonstrate that DSC-DeepLabv3+ strikes an effective balance between accuracy and efficiency, outperforming several classical lightweight models, making it a promising solution for accurate and efficient weed segmentation in agricultural applications.

## 1 Introduction

Weeds negatively impact crop yield (Moreau et al., 2022) and quality (Hamuda et al., 2016), making effective weed control essential in crop management. Although conventional herbicides are widely used in maize fields due to their ease of application and effectiveness (Zou et al., 2021), they may they may harm soil health, negatively affect maize, and threaten human health (Muola et al., 2021). In recent years, weed recognition systems based on machine vision have made progress. However, traditional machine learning methods such as ANN (Shah et al., 2021), naive Bayes, decision tree, K-means (Agarwal et al., 2021), and support vector machine (SVM) (Zhang et al., 2022a) are highly sensitive to environmental variation, which limits their robustness and applicability in precision agriculture (Espejo-Garcia et al., 2020). With the rapid development of deep learning, convolutional neural networks have demonstrated strong feature learning capabilities (Fuentes-Pacheco et al., 2019). In particular, semantic segmentation has emerged as a mainstream technique for weed identification (Subeesh et al., 2022). However, popular models such as FCN (Shelhamer et al., 2017), Unet (Ronneberger et al., 2015), DeepLab series (Chen et al., 2016, 2018b; Sandler et al., 2018), and PSPNet (Zhao et al., 2017) are characterized by large parameter sizes, high computational costs, and low inference speed, which limit their practical application and segmentation performance in real-world agricultural settings. To address these limitations, researchers have proposed various lightweight semantic segmentation models designed for real-time applications.

In lightweight semantic segmentation, simplifying the model architecture is a primary objective. Enet (Paszke et al., 2016) introduced an asymmetric encoder–decoder structure, significantly reducing parameter and memory costs and laying the foundation for real-time semantic segmentation. ERFNet (Romera et al., 2018), EACNet (Li et al., 2021), and LMFFNet (Shi et al., 2023) built upon this structure, further reducing parameters through factorized convolutions. The success of SegNet (Badrinarayanan et al., 2017) demonstrated the effectiveness of encoder–decoder architectures with skip connections in resource-constrained environments. DABNet (Li et al., 2019) and CGNet (Wu et al., 2021) utilized dilated convolutions to capture both local and contextual features, thereby improving segmentation accuracy. LEDNet (Wang et al., 2019) and LAANet (Zhang et al., 2022b) incorporated attention mechanisms to enhance contextual feature representation and boost performance. The ICNet (Zhao et al., 2018) adopted a cascaded multi-resolution structure to balance segmentation accuracy and real-time efficiency. BiSeNet (Yu et al., 2018) introduced a dual-branch architecture to separately extract spatial and semantic features. BiSeNetV2 (Yu et al., 2021) and STDC (Fan et al., 2021) further optimized efficiency through feature-sharing designs. Although PIDNet (Xu et al., 2023) and DDRNet (Pan et al., 2023) improved performance by employing multi-path strategies, they introduced additional computational burdens. Recent trends in lightweight segmentation focus on innovative architectures, efficient feature fusion modules, and weakly or unsupervised learning methods to enhance adaptability and performance.

In recent years, lightweight semantic segmentation algorithms have shown great potential in practical applications. In the field of crop–weed and remote sensing image segmentation, Zuo et al (Zuo and Li, 2024). proposed a lightweight U-Net variant, which enhances cornfield weed segmentation efficiency by incorporating an inverted residual structure, pyramid pooling, and a squeeze-and-excitation mechanism. Sun et al (Sun et al., 2025). proposed ASLMSHNet, which optimizes feature fusion and resource allocation for remote sensing image segmentation through progressive dilated convolutions, adaptive sparse cross-attention, and multi-scale feature alignment. Janneh et al (Janneh et al., 2023). introduced a multilevel feature reweighting framework that comprises a lightweight backbone, a reweighting fusion module, and a convolutional weighted decoder. This approach reduces feature dimensionality, suppresses background interference, and improves both contextual understanding and segmentation efficiency. In the domain of plant disease and infrastructure defect detection, Feng et al (Feng et al., 2022). introduced DFFANet, which integrates deep feature fusion and attention mechanisms through modules such as DCABlock, FFM, and an efficient attention module. This design ensures accurate segmentation of rice blast spots while maintaining low model complexity. Yu et al (Yu et al., 2025). improved the DeepLabv3+ model for bridge deck disease detection by incorporating MobileNetV3, a CSF-ASPP module, and a focal loss function. These modifications significantly reduced parameter count and computational complexity while enhancing the recognition accuracy of small-scale disease regions. While these models demonstrate strong performance in specific scenarios, lightweight networks often suffer from accuracy degradation when reducing parameters or accelerating inference. Moreover, most existing methods are tailored to specific domains, limiting their generalization and adaptability across diverse field conditions. In addition to architectural improvements, recent studies have explored optimization strategies at the feature level to further enhance model performance. For instance, Xie et al (Xie et al., 2023). proposed a feature selection strategy based on the Salp Swarm Algorithm for plant disease detection. Such approaches highlight the potential of bio-inspired algorithms in reducing model complexity while maintaining performance.

We chose the DeepLabv3+ (Chen et al., 2018) model as the basic framework due to its excellent performance in semantic segmentation. However, the model has several limitations: its feature extraction network is overly complex and contains a large number of parameters. Additionally, the use of standard convolutions in the ASPP module further increases the parameter count, thereby increasing model complexity, hardware requirements, and reducing training efficiency. Moreover, the encoder stage progressively reduces the spatial resolution of the input, leading to information loss and insufficient restoration of fine details during decoding. As a result, boundary localization remains suboptimal. Although the ASPP module enhances boundary extraction, it fails to adequately model local feature relationships, leading to fragmented segmentation and reduced accuracy, particularly at object edges. To address these limitations and achieve improved accuracy, lightweight design, and faster inference, we propose an enhanced version of DeepLabv3+. The primary contributions of this study are summarized as follows:

1. The original network has been substituted using MobileNetV2, while standard convolutions in the encoder-decoder segments were substituted with depthwise separable dilated convolutions, thereby reducing computational load and training time.
2. To accurately capture distant dependencies and acquire dense contextual information, the strip pooling is integrated within the ASPP module, and CBAM is applied after ASPP to enhance feature maps’ capacity to extract detailed information.
3. To fully utilize features from the two intermediate layers and improve segmentation accuracy, in the decoder part, we propose the C-CFF module for feature fusion.

## 2 Materials and methods

### 2.1 Construction of the maize weed dataset

#### 2.1.1 Data acquisition

Maize weed images were collected at Jilin Agricultural University (Changchun, Jilin Province, China) between 10:30 and 14:30 on June 10 and June 20, 2024. Data were captured using a Xiaomi 14 smartphone equipped with a 50 MP rear camera (ISO 50, shutter speed: 1/200 s), mounted vertically at a height of 50 cm above the ground. Videos with a resolution of 1280 × 720 pixels were recorded and subsequently converted into individual JPG images of the same resolution.

#### 2.1.2 Data preprocessing

**Figure 1:**
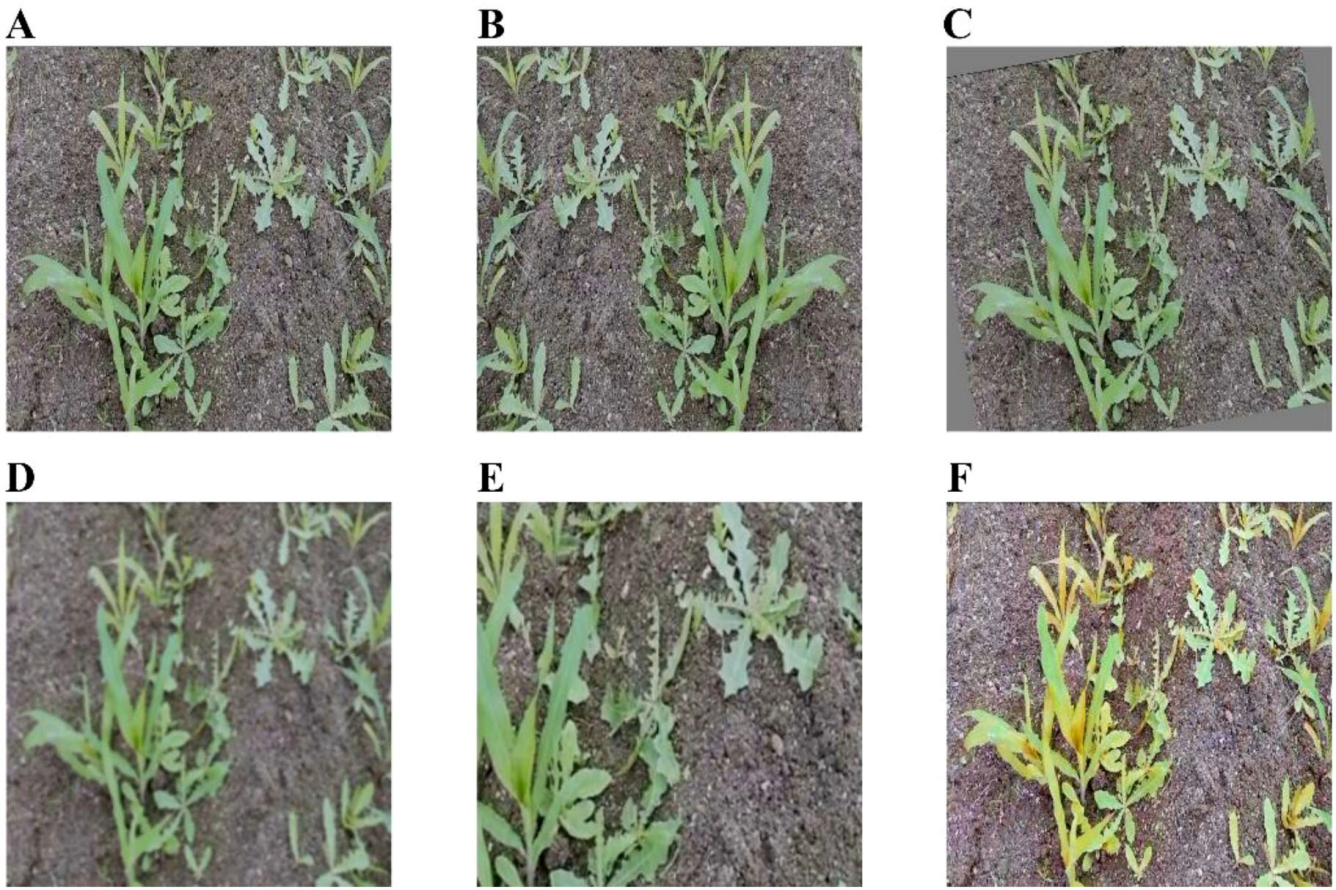
Examples of data augmentation. **(A)** Original image. **(B)** Flip. **(C)** Rotation. **(D)** Gaussian Blur. **(E)** Random Crop. **(F)** HSV.

After removing unusable samples, a final set of 481 valid images containing both maize seedlings and weeds was retained. These images were subsequently annotated using LabelMe (Russell et al., 2008), with each pixel classified into three categories: “corn” for maize seedlings, “weed” for weeds, and background for all other regions. Data augmentation was employed to enhance the model’s robustness, with the specific techniques illustrated in **Figure 1**. Following augmentation, the dataset was expanded to 2,886 images, ensuring sufficient diversity for training and reliable performance evaluation. The dataset was randomly split into training and validation subsets at a 9:1 ratio, and all images were subsequently converted to Pascal VOC format for use in this study.

### 2.2 DeepLabv3+ model structure

**Figure 2:**
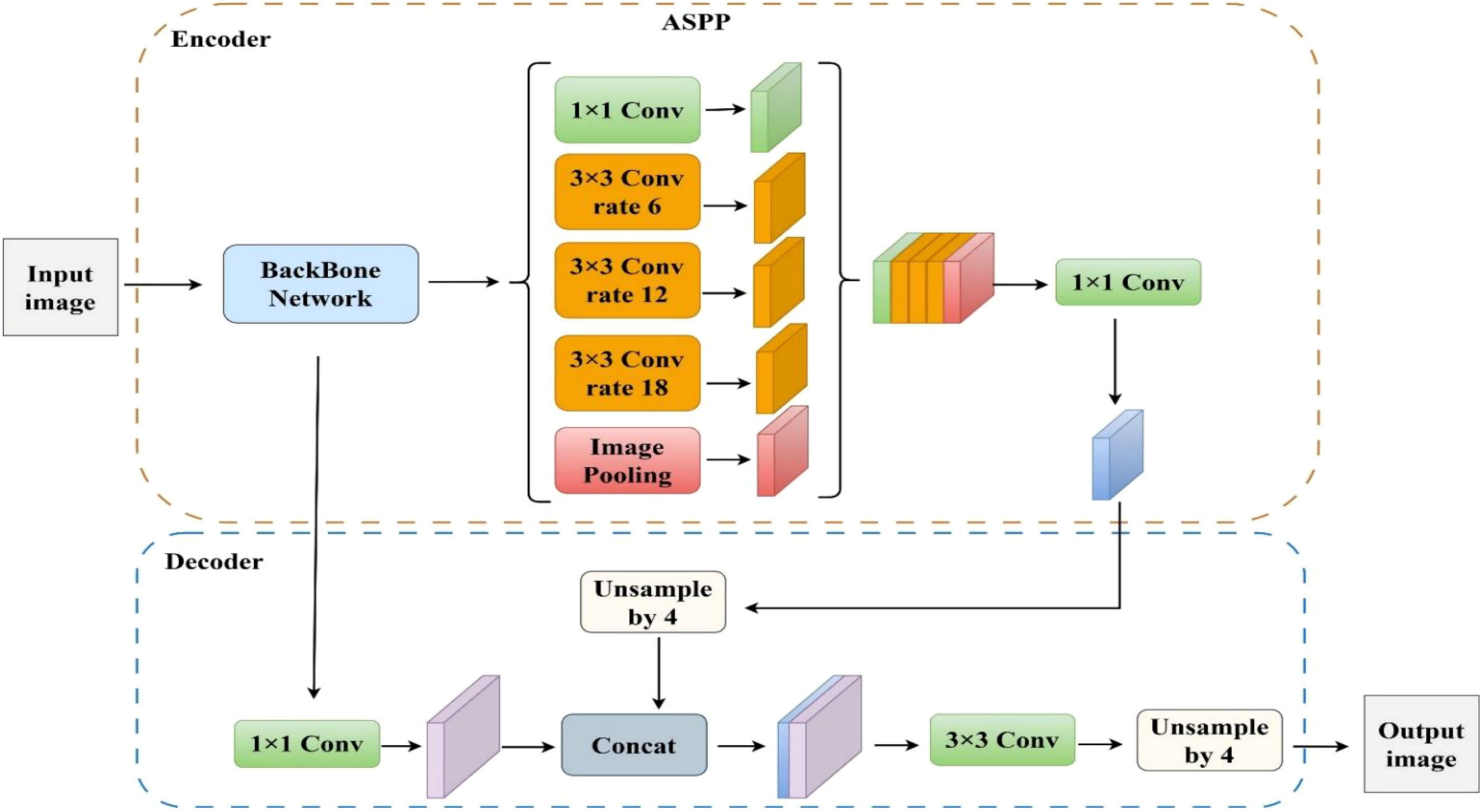
The original DeepLabv3+ model structure.

As shown in **Figure 2**, DeepLabv3+ is a semantic segmentation model developed by Google that employs an encoder–decoder architecture to integrate multi-scale contextual features and fine spatial details. It uses the Xception network (Chollet, 2017) as its feature extraction backbone and integrates an ASPP module, which combines a 1×1 convolution with three parallel 3×3 dilated convolutions at different dilation rates to capture multi-scale contextual information. The decoder fuses high-level semantic features with low-level edge information, enabling accurate pixel-wise segmentation and enhancing boundary delineation in small objects and complex scenes.

### 2.3 Improved DeepLabv3+ semantic segmentation model

**Figure 3:**
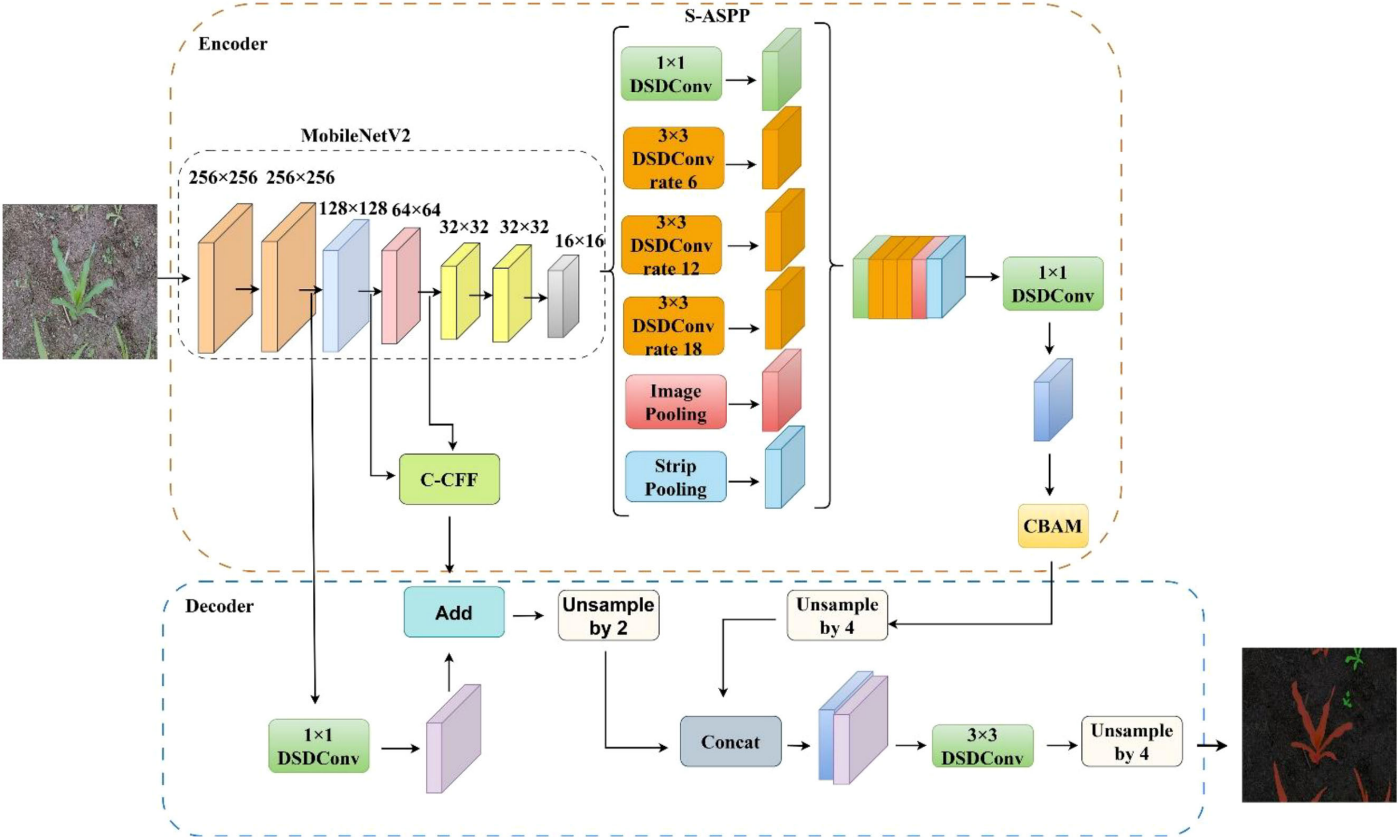
Improved DSC-DeepLabv3+ model structure.

Although DeepLabv3+ exhibits strong performance in semantic segmentation, certain limitations remain that hinder its broader applicability. The encoder reduces the spatial resolution of feature maps and fails to fully exploit high-resolution information, leading to discontinuities in predictions and a loss of fine-grained details. While the ASPP module enhances boundary feature extraction, it inadequately captures local structural information, particularly for radially distributed objects such as weeds and maize leaves, resulting in fragmented segmentation and semantic gaps. Moreover, the Xception backbone introduces a substantial computational burden, increasing hardware demands and reducing training efficiency. To address these limitations, this study proposes a lightweight improved DSC-DeepLabv3+ model that optimizes the encoder–decoder architecture and enhances both computational efficiency and segmentation accuracy. An overview of the proposed model is illustrated in **Figure 3**. In the encoder, the original Xception backbone is replaced with MobileNetV2 to achieve efficient feature extraction. The ASPP module is enhanced with depthwise separable convolutions to reduce parameters and improve training efficiency. To further enrich global and local contextual information, a strip pooling module is integrated, resulting in a modified S-ASPP structure with six parallel branches. Additionally, the CBAM is incorporated to enhance segmentation accuracy while maintaining low computational complexity. On the decoder side, the C-CFF module is employed to fuse 1/8 and 1/16 scale feature maps extracted from the backbone. CBAM is introduced again to suppress redundant noise and alleviate edge blurring. Shallow features are fused and upsampled via bilinear interpolation to restore spatial details. The fused deep and multi-scale features are subsequently processed through a 3×3 convolution and fourfold upsampling to restore the original image resolution.

### 2.4 Backbone Network

**Figure 4:**
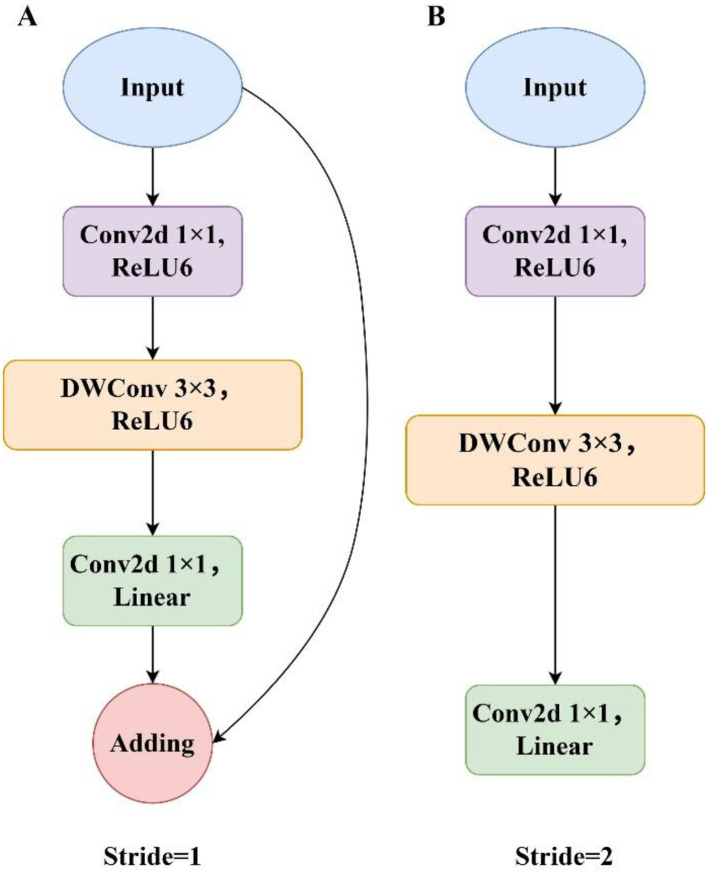
The inverted residual and linear bottleneck structure. **(A)** Inverted residual block with a stride of 1. **(B)** Inverted residual block with a stride of 2.

The original DeepLabv3+ employed the Xception network as its feature extraction backbone. Although effective on large-scale datasets, its performance degrades in practical applications characterized by limited annotated data. Its large parameter count and complex architecture result in slow inference and extended training time, hindering deployment on mobile and embedded platforms. To address these limitations, this study proposes a lightweight model by adopting MobileNetV2 (Sandler et al., 2018) as the backbone. MobileNetV2 was introduced by Google in 2018, featuring a streamlined architecture optimized for mobile and embedded devices. It was designed to offer an efficient solution for various visual recognition tasks, including object classification and image segmentation. The architectural details of the backbone network are presented in **Table 1**. The core innovation of MobileNetV2 lies in replacing standard convolutions with depthwise separable convolutions. This approach significantly reduces computational cost and model size by decomposing standard convolutions into two operations: depthwise convolution and pointwise convolution. In this structure, the depthwise operation applies a 3×3 convolution to each input channel independently, while the pointwise operation performs a 1×1 convolution to aggregate information across channels and produce the final feature map. This design improves training efficiency and reduces the computational cost of 3×3 convolutions by up to 90%, while maintaining high segmentation accuracy. However, despite these improvements, feature extraction remains suboptimal due to kernel sparsity, with a large proportion of convolutional parameters contributing minimally. To address these issues, MobileNetV2 introduces the linear bottleneck and inverted residual structure, as illustrated in **Figure 4**. The linear bottleneck reduces parameter count and computational overhead by eliminating nonlinear activations that may distort low-dimensional feature representations. The inverted residual structure, incorporating designs with two different strides, facilitates network deepening, prevents the vanishing gradient problem, and decreases the number of parameters. Collectively, these innovations allow MobileNetV2 to achieve high performance while maintaining low computational complexity.

**Table 1:** The structure of the backbone.

Input	Operator	t	c	n	s​
512×512×3	conv2d		32	1	2
256×256×32	Bottleneck	1	16	1	1
256×256×16	Bottleneck	6	24	2	2
128×128×24	Bottleneck	6	32	3	2
64×64×32	Bottleneck	6	64	4	2
32×32×64	Bottleneck	6	96	3	1
32×32×96	Bottleneck	6	160	3	2
16×16×160	Bottleneck	6	320	1	1
16×16×320	conv2d		1280	1	1
16×16×1280	AvgPool		1280	1	
1×1×1280	Classifier		k		

t, is the expansion factor in Bottleneck; c, is the number of output channels; n, is the number of repetitions of the operation; s, is the step size.

### 2.5 S-ASPP

**Figure 5:**
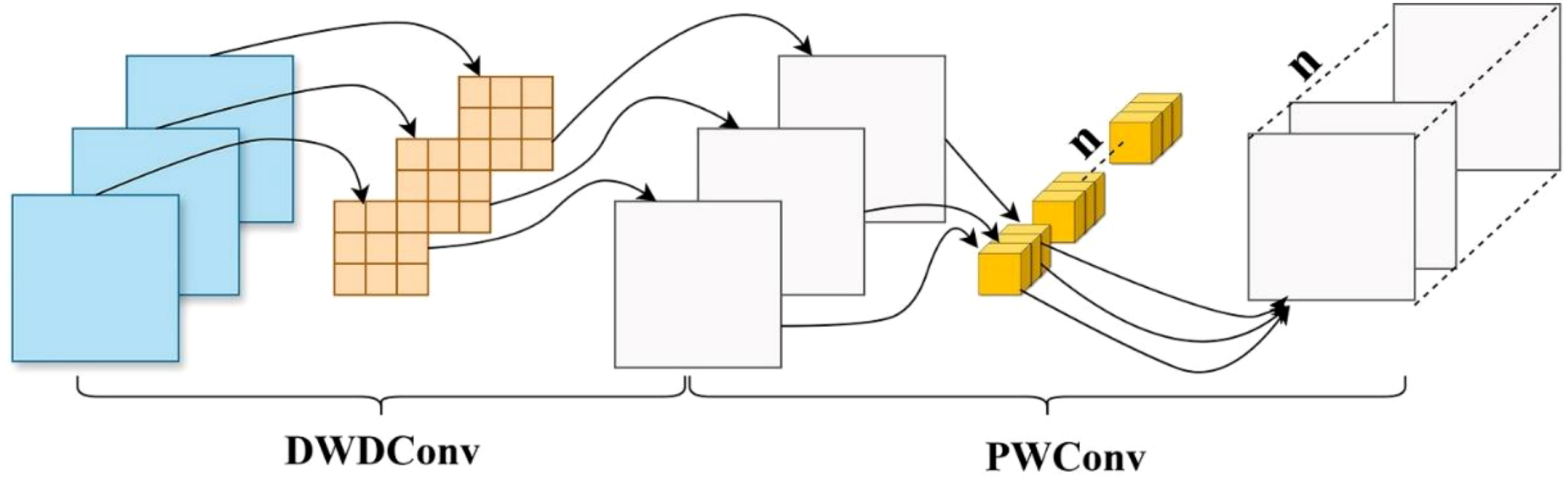
The structure of depthwise separable dilated convolution.

**Figure 6:**
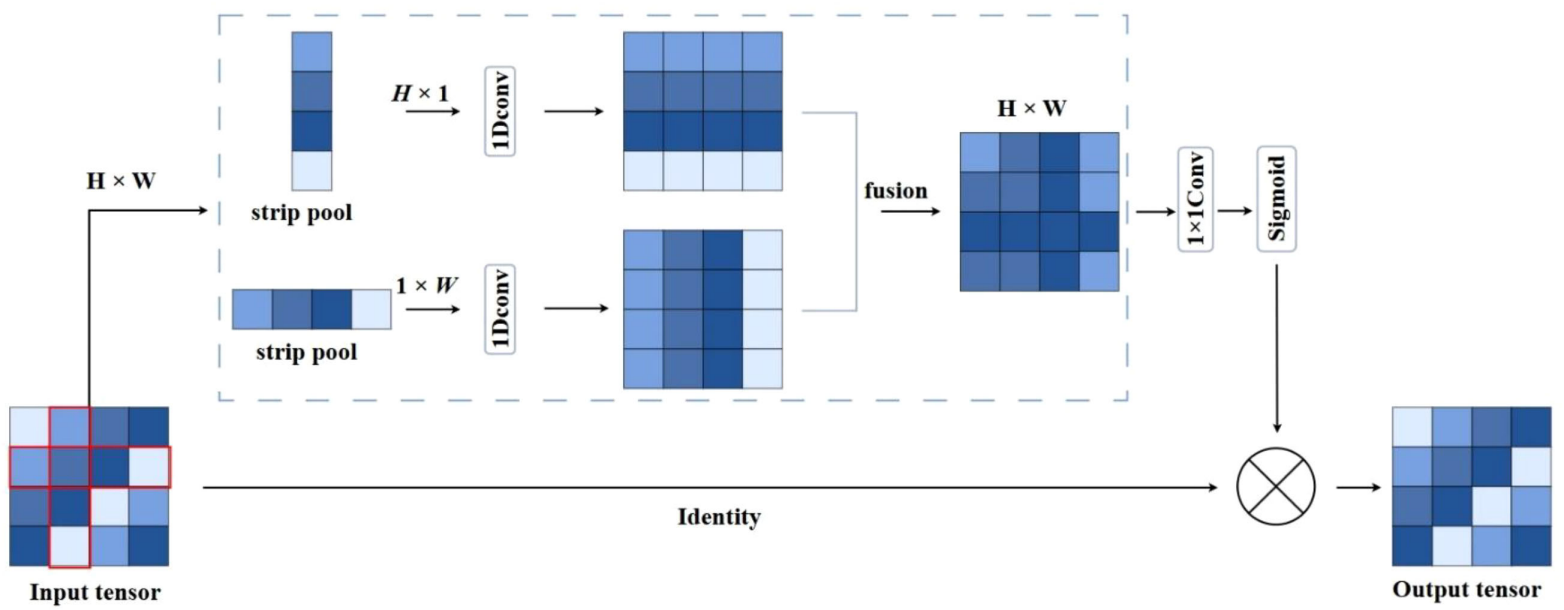
The structure of strip pooling.

This study enhances training efficiency by introducing depthwise separable dilated convolutions, which integrate the benefits of both dilated and depthwise separable operations, as illustrated in **Figure 5**. In the depthwise stage, each channel of the feature map is convolved independently with a dilated kernel to extract spatial correlations while preserving local structural information. Subsequently, a 1×1 pointwise convolution aggregates the features across channels to generate the final output. This method enlarges the receptive field, reduces parameter count and computational complexity, and accelerates inference. Additionally, the global average pooling in the traditional ASPP (He et al., 2015) employs a fixed-size square window, which presents certain limitations. Such a design struggles to capture directional scale correlations when processing irregularly shaped objects or complex environments. The square pooling window may introduce redundant dependencies and incorporate noise from unrelated regions, leading to the loss of critical fine-grained information. To overcome these limitations, Strip Pooling (Hou et al., 2020) is integrated into the ASPP module, resulting in a novel six-branch S-ASPP structure. This modification reduces model complexity, enhances inference speed, and enables the capture of diverse multi-scale contextual features. Strip pooling performs directional pooling along horizontal and vertical dimensions to simultaneously capture global and local contextual information while suppressing background noise. It utilizes one-dimensional convolutions along each direction within a residual framework to enhance directional sensitivity. The resulting feature maps are fused and element-wise multiplied with the original features to refine spatial representations. Unlike traditional square pooling, strip pooling independently processes vertical and horizontal spatial dimensions by performing weighted averaging across rows and columns. The structure of the strip pooling module is illustrated in **Figure 6**.

For the given input image, the two vectors of the input picture are computed using Equations 1, 2:


(1) $$y_{i}^{h}=\frac{1}{W}\sum_{j=0}^{W}x_{i,j}$$

(2) $$y_{j}^{v}=\frac{1}{H}\sum_{i=0}^{H}x_{i,j}$$

For an input $X\in {\mathbb{R}}^{C\times H\times W}$ , the total amount of channels is denoted by C,i,j denotes the row and column respectively, H,W represents the size, X is passed via both vertical and horizontal paths for pooling. The horizontal and vertical outputs are $y^{v}\in {\mathbb{R}}^{C\times H\times W}$ . Following their combination, the Equation 3 is used to figure out the final output:


(3) $$y_{c,i,j}=y_{c,j}^{h}+y_{c,j}^{v}$$

A refined feature map is obtained after completing the convolution operations and applying the sigmoid activation function, and then it is merged with the input feature map for the production of z . The specific process is shown in Equation 4:


(4) z=Scale(X,σ(f(y)))

### 2.6 Improved C-CFF module

#### 2.6.1 CFF module

The original Cascade Feature Fusion (CFF) module in ICNet (Zhao et al., 2018) improved semantic segmentation by fusing multi-resolution features to enhance spatial detail preservation. Specifically, it accepts a shallow feature map F_1_ and a deep feature map F_2_ as inputs. First, F_2_ is upsampled by a factor of two using bilinear interpolation, followed by a dilated convolution to achieve spatial alignment with F_1._ A 1×1 convolution is then applied to F_1_ to adjust its channel dimensions to match those of the processed F_2._ Both feature maps are normalized through batch normalization layers, then fused via element-wise addition. The resulting map is passed through a ReLU activation function to obtain the final fused feature map F_c_. However, the simple element-wise addition disregards the heterogeneity in semantic and spatial information between the features, which limits its effectiveness in tasks requiring fine-grained segmentation, such as leaf edge and small object recognition. Furthermore, this naive fusion strategy may introduce redundant noise across both channel-wise and spatial dimensions. In complex agricultural scenarios that demand precise localization of crop and weed boundaries, the original CFF module lacks the capacity to emphasize salient regions, potentially leading to blurred boundaries and loss of detailed features.

#### 2.6.2 CBAM

**Figure 7:**
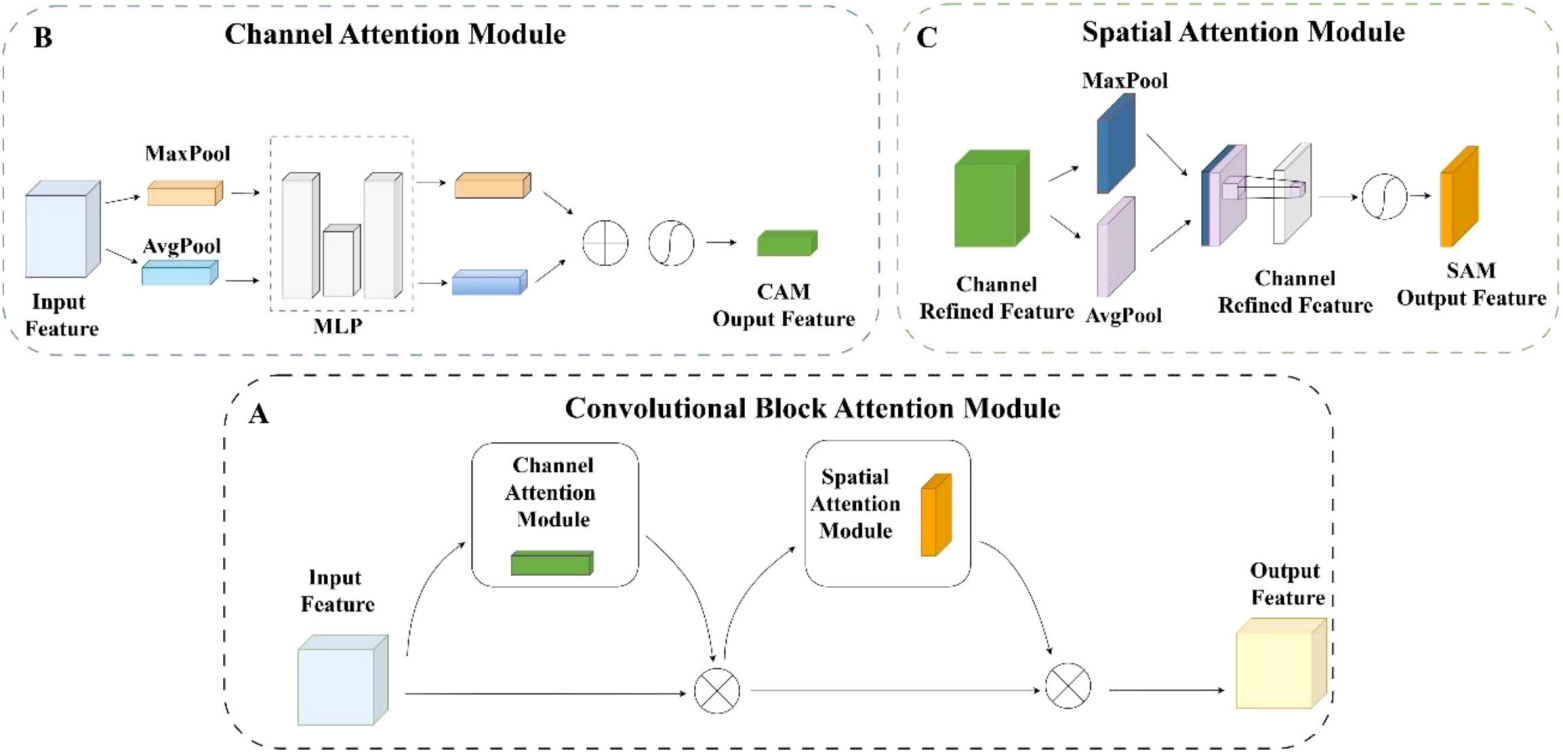
**(A)** Convolutional block attention module. **(B)** Channel attention module. **(C)** Spatial attention module.

In recent years, attention mechanisms have become widely used in computer vision tasks due to their ability to selectively focus on salient regions and efficiently capture informative visual cues. Such mechanisms have been increasingly integrated into convolutional neural networks to enhance performance in large-scale image classification tasks. CBAM (Woo et al., 2018) is a lightweight attention module that combines both channel and spatial attention to significantly improve model accuracy while introducing minimal computational overhead. It can be seamlessly embedded into various convolutional neural network architectures without requiring extensive modifications. As illustrated in **Figure 7**, CBAM consists of two sequential submodules. It generates attention maps by analyzing intermediate feature representations to emphasize salient features and suppress irrelevant information, thereby improving the network’s ability to extract meaningful patterns from complex visual data.

This channel attention part is designed based on treating every channel as a distinct feature detector. Specifically, spatial information within representations of features is first compressed using two global pooling processes, resulting in channel attention representations. Vectors are passed through an MLP, resulting in two attention vectors of size C×1×1 . By element-wise summing the outputs and applying a sigmoid activation function, a final channel attention vector $M_{c}$ of size C×1×1 is obtained. Its calculation formula is shown in Equation 5:


(5) $$M_{c}(F)=\sigma \left(\omega F_{\text{max}}^{c}+\omega F_{\text{avg}}^{c}\right)$$

where F is the given middle feature, c denotes the channel dimension,avg denotes global average pooling, max denotes maximum pooling, σ denotes Sigmoid, and ω represents the fully connected operation.

#### 2.6.3 CBAM-cascade feature fusion module

**Figure 8:**
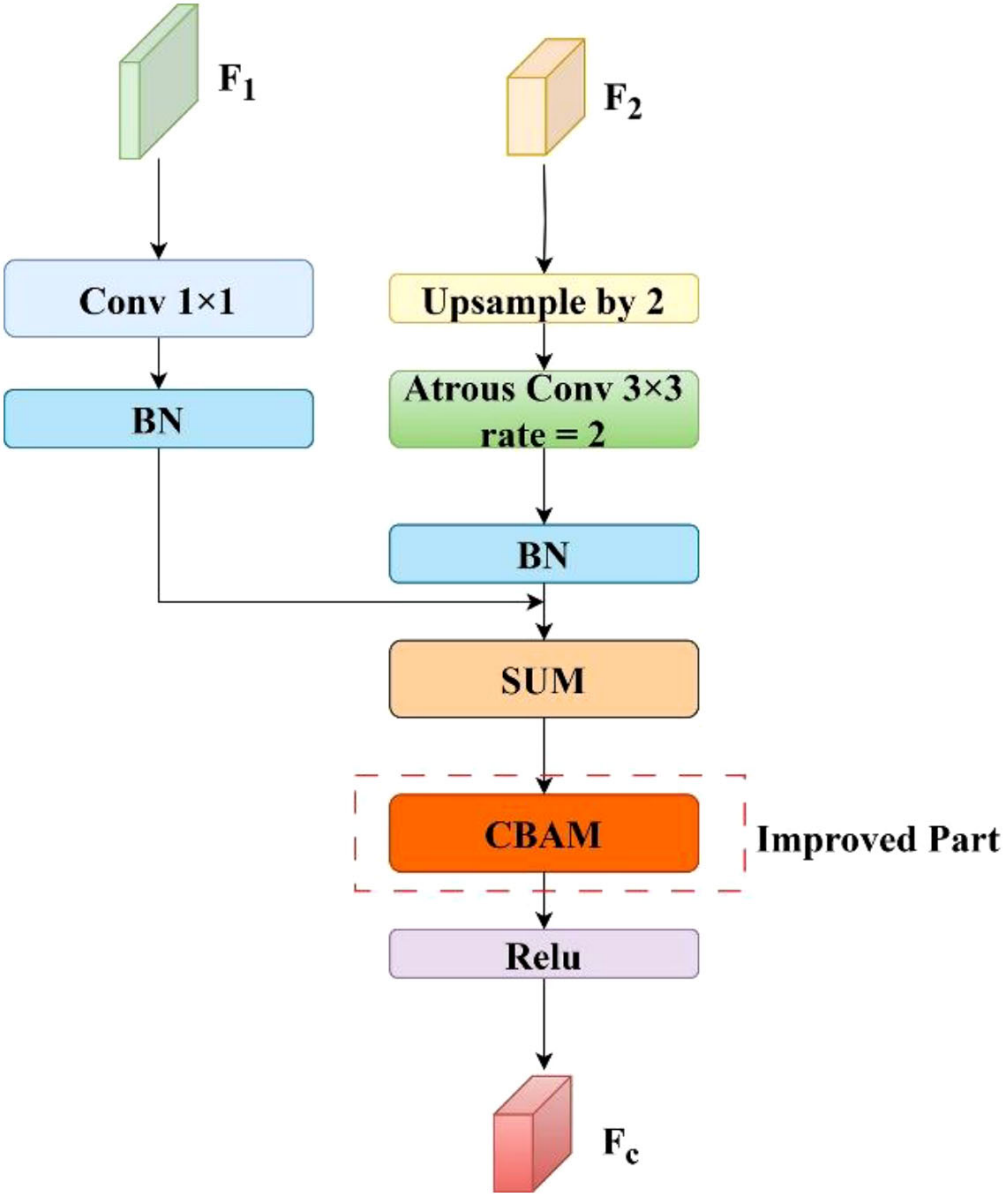
Structure of the improved C-CFF module.

Motivated by the effectiveness of attention mechanisms in enhancing feature selection, we incorporated the CBAM module into the CFF structure. Specifically, CBAM is positioned after the fusion of the feature maps F_1_ and F_2_, enabling refined recalibration of the fused features. It adaptively recalibrates the importance of each semantic channel, thereby enhancing the representation of fine-scale targets such as crop seedlings. Moreover, its spatial attention branch emphasizes high-frequency regions such as leaf boundaries, improving the network’s capacity to capture fine-grained spatial details. This strategy suppresses background interference while enhancing the boundary sensitivity of the segmentation map. As a result, the network dynamically balances feature responses along both channel and spatial dimensions, facilitating accurate boundary recovery without compromising computational efficiency. The architecture of the enhanced fusion module is illustrated in **Figure 8**. This improved feature fusion approach preserves shallow feature richness while fully utilizing deep semantic representations, thereby enhancing both the segmentation accuracy and the robustness of the network.

For feature maps $F_{1}^{C_{1}\times H_{1}\times W_{1}}$ and $F_{2}^{C_{2}\times H_{2}\times W_{2}}$ , where the size of F_1_ is twice that of F_2_. We first apply an upsampling rate of 2 on F_2_ through bilinear interpolation, followed by dilated convolution to keep same as F_1_. Meanwhile, F_1_ conducts a 1x1 convolution operation to achieve the same number of feature channels as F_2_. These two processed features are then normalized using two batch normalization layers. Finally, the two features were added together to obtain F_3_ as described by Equation 6:


(6) $$F_{3}=\beta (\kappa (F_{1}))+\beta \left(\phi \left(\gamma (F_{2})\right)\right)$$

where κ denotes a 1 × 1 convolution, ϕ is a dilation convolution, γ denotes upsampling, and β denotes batch normalisation.

Subsequently processed by the CBAM module, the channel attention weights were first generated by the channel attention module $M_{c}$ . The channel attention map G is obtained by multiplying this weight by the input features. Then, the spatial attention module was used to generate the spatial attention weights $M_{s}$ and get the spatial attention feature map τ , which is finally activated by the ReLU and generates the final output $F_{c}$ . Specific calculation steps are calculated via Equations 7–9:


(7) $$G=M_{c}⊙F_{3}=\sigma \left(\omega F_{\text{max}}^{c}+\omega F_{\text{avg}}^{c}\right)⊙\beta \left(\kappa (F_{1})\right)+\beta \left(\phi \left(\gamma (F_{2})\right)\right)$$

(8) $$\tau (G)=M_{s}\left(M_{c}⊙F_{3}\right)⊙\left(M_{c}⊙F_{3}\right)$$

(9) $$F_{c}=\delta \left(\tau (G)\right)$$

where ⊙ denotes element-wise multiplication, G denotes the feature after channel attention processing, τ denotes the spatial attention feature map after processing, and δ denotes the ReLU activation function.

## 3 Results

### 3.1 Evaluation metrics

The purpose of this paper is to keep a lightweight model while obtaining outstanding segmentation accuracy. We evaluate the model’s performance using widely utilized semantic segmentation metrics such as mIoU. The calculation of the mIoU is defined by Equation 10:


(10) $$\text{mIoU}=\frac{1}{n}\sum_{i=1}^{n}\frac{\text{TP}}{\text{TP}+\text{FP}+\text{FN}}\times 100\%$$

where n is the number of classes, TP is true positive, TN is true negative, FP is false positive, and FN is false negative.

Parameters reflect the size of the model. FLOPs (Floating Point Operations) are its computational complexity. FPS measures processing speed, representing the time taken to process a picture.

### 3.2 Model training

The semantic segmentation model was implemented in the PyTorch framework under the following software environment: Python 3.8.19, PyTorch 2.4.0, CUDA 11.8, and Windows 11. The experiments were conducted on a workstation equipped with an AMD 7745HX CPU and an NVIDIA GeForce RTX 4060 GPU.

During training, input images were resized to 512×512 pixels. Stochastic Gradient Descent (SGD) optimizer was adopted as the optimizer, with an initial learning rate of 0.007, a minimum learning rate set to 0.01 of the maximum, and a weight decay of 0.0001. The training process lasted for 300 epochs, with the first 100 epochs conducted under a frozen backbone using a batch size of 8, and the remaining 200 epochs under an unfrozen backbone with a batch size of 4. Model validation and checkpoint saving were performed every 20 epochs. In the dataset, maize occupies the majority of the pixel area, resulting in significant foreground-background class imbalance. To mitigate the adverse effects of this imbalance on segmentation performance, the cross-entropy loss function was employed, as defined in Equation 11:


(11) $$\text{Cross\_entropy}=-\frac{1}{N}\sum_{i}\sum_{c=1}^{M}y_{ic}\log(p_{ic})$$

where N is the overall count of samples, M symbolizes how many classes, $y_{ic}$ is the true value of the i-th sample belonging to class c, and $p_{ic}$ is the model’s projected likelihood that the i-th sample falls into class c.

**Figure 9:**
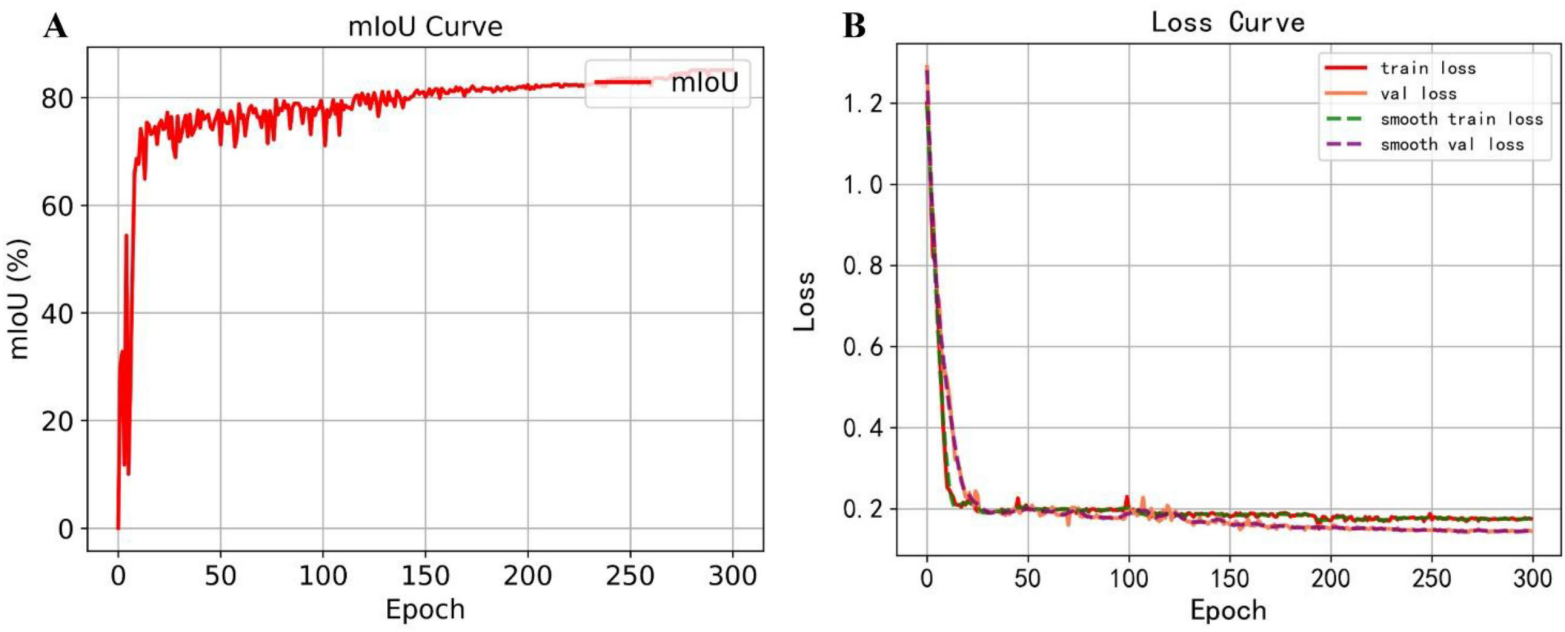
Model training results. **(A)** Training mIoU change curve. **(B)** Training loss curve.

Thanks to the pre-trained backbone adopted through transfer learning, the training loss converged rapidly to a low value. **Figure 9** depicts the mIoU and loss curves of the proposed model. After 300 epochs, the mean Intersection over Union (mIoU) reached 85.47%. As shown in the loss curve, the validation and training losses decreased to approximately 0.1 and 0.2, respectively, with minimal fluctuations, indicating stable convergence. Beyond this point, further training yielded marginal improvements in loss, suggesting that the model had reached optimal convergence.

### 3.3 Comparison of various models for semantic segmentation

**Figure 10:**
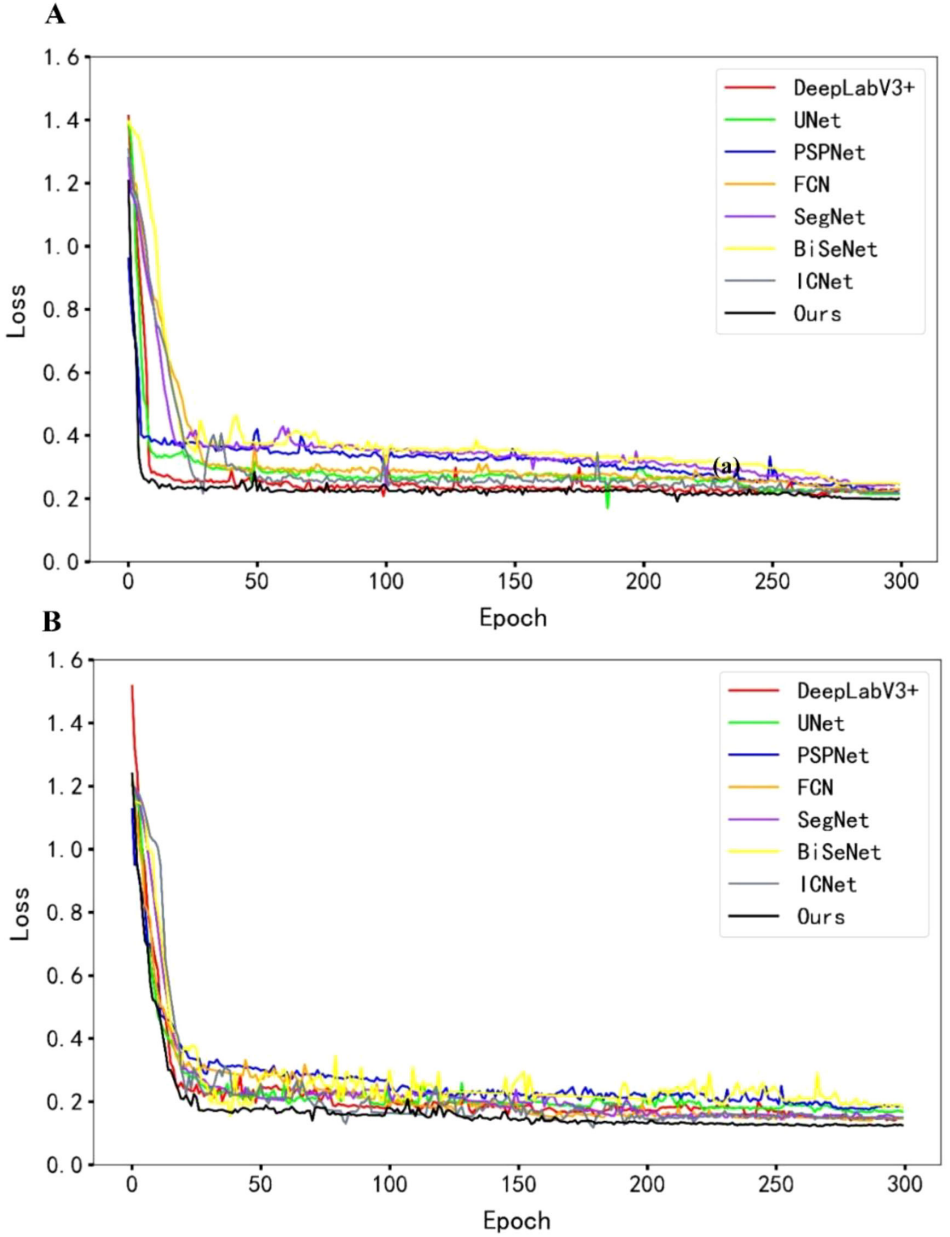
Loss comparison plots. **(A)** Training loss plots. **(B)** Validation loss plots.

To evaluate the performance of the proposed method in maize field weed recognition, we compared it with both classical and lightweight models, including SegNet, BiSeNet, and ICNet. All models were trained under identical experimental conditions and preprocessing procedures. As shown by the loss curves in **Figure 10**, the proposed model demonstrates a faster convergence rate and achieves lower final loss values within 300 training epochs for both the training and validation sets. Compared to other models, it also shows a steeper initial loss descent and faster overall convergence, effectively reducing the required training time.

This study compares the accuracy and computational complexity of various models, as summarized in **Table 2**. The proposed model reduced FLOPs to 15.326G, which is approximately 150G less than the original DeepLabv3+, while incurring only a 0.71% decrease in mean Intersection over Union (mIoU). Its parameter count was reduced to just 5% of the original, and inference speed increased by 24.49 FPS, making it well-suited for mobile deployment. Compared to SegNet and ICNet, the proposed model achieved mIoU improvements of 7.67% and 5.92%, respectively. It also demonstrated notable gains in inference speed (33.44 and 12.59 FPS), while reducing parameters by 26.55M and 23.61M, and FLOPs by 111G and 12.974G. Compared to BiSeNet, it achieved slightly higher inference speed and a 7.1% increase in mIoU, striking a balance among model compactness, computational efficiency, and segmentation performance.

**Table 2:** Comparison of segmentation results of several methods.

Model	Backbone	mIoU (%)	Parameters (M)	FLOPs (G)	FPS
FCN	VGG16	75.0	32.75	89.8	14.73
Unet	VGG16	79.2	43.93	184.4	27.76
PSPNet	ResNet50	74.7	46.716	118.47	29.4
DeepLabv3+	Xception	**86.28**	54.714	167.139	18.4
SegNet	VGG16	77.9	29.44	126.34	9.45
BiSeNet	Xception	78.47	5.8	50.3	41.23
ICNet	ResNet50	79.65	26.5	28.3	30.3
Ours	MobileNetV2	85.57	**2.89**	**15.326**	**42.89**

Performance comparison of different models. Bold values indicate the best results.

**Figure 11:**
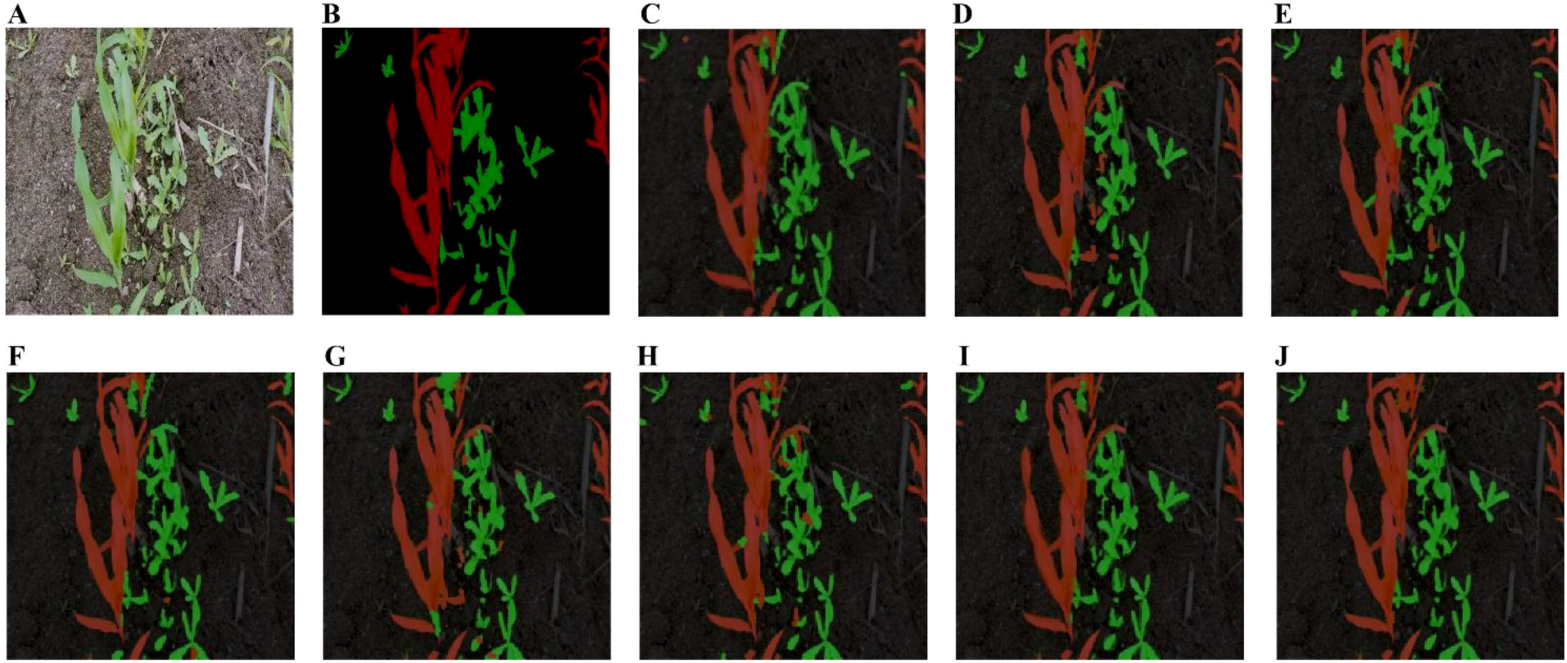
Segmentation results of various models on the maize weed dataset. **(A)** Original image. **(B)** Label image. **(C)** Unet. **(D)** FCN. **(E)** PSPNet; **(F)** SegNet; **(G)** BiseNet; **(H)** ICNet; **(I)** DeepLabv3+; **(J)** Ours.

**Figure 11** presents the segmentation results of various models on the maize weed dataset. The proposed model demonstrates superior performance in boundary delineation and pixel-level classification, particularly in scenarios involving overlapping maize and weeds with incomplete or ambiguous shape features. In contrast, models such as PSPNet, FCN, BiSeNet, and ICNet exhibit misclassification of adjacent pixels, primarily due to insufficient global contextual modeling. For instance, the pyramid pooling module in PSPNet compromises spatial detail, while FCN’s limited receptive field overly emphasizes local features, leading to errors in blurred boundary regions. Although BiSeNet and ICNet achieve faster inference through multi-branch architectures, their aggressive downsampling and coarse feature fusion reduce semantic consistency, particularly affecting sensitivity to small objects. U-Net and DeepLabv3+ also suffer from imprecise edge segmentation. In U-Net, skip connections introduce noise from shallow layers, which, when fused with deep features, contribute to edge blurring. Standard upsampling operations further smooth the boundaries, undermining the recovery of fine-grained details. Although the ASPP module in DeepLabv3+ expands the receptive field, its reliance on standard convolutions and global pooling reduces responsiveness to high-frequency edge features. Additionally, the lack of targeted enhancement mechanisms during low-resolution feature fusion in the decoder leads to boundary deviations from the actual object contours. In contrast, the proposed model incorporates strip pooling to capture long-range contextual dependencies and mitigate pixel misclassification in overlapping regions. Furthermore, a feature fusion module enhances the recovery of essential spatial details for precise boundary localization. This architecture effectively balances segmentation accuracy and model efficiency, making it highly suitable for real-time maize weed recognition in complex field environments.

### 3.4 Ablation experiments

#### 3.4.1 Ablation experiments of the C-CFF module

To assess the effectiveness of the improved C-CFF module, we conducted ablation studies using a controlled variable approach. As shown in **Table 3**, incorporating either CAM or SAM individually led to slight increases in parameters and FLOPs, yielding mIoU improvements of 0.53% and 0.65%, respectively. By contrast, integrating CBAM into the CFF module improved mIoU by 1.51% over the baseline, with only a modest increase of 0.7M parameters and 0.53 GFLOPs. Although the 7×7 convolution in the spatial attention mechanism slightly reduced the inference speed, the overall computational overhead remained minimal. These findings validate the effectiveness of the C-CFF module in enhancing segmentation performance. However, the relatively limited improvements achieved by CAM and SAM individually warrant further analysis. A possible explanation is that, after multiple layers of convolution and pooling, the deep feature maps already contain abundant semantic information. In such scenarios, employing a single channel or spatial attention mechanism may fail to extract additional meaningful features, potentially resulting in redundancy and misaligned attention. Moreover, CAM and SAM were integrated independently into the CFF module without interaction, which limited their overall effectiveness. In contrast, CBAM sequentially applies channel and spatial attention, first highlighting informative channels and then emphasizing critical spatial regions. This sequential mechanism enhances feature selection more effectively. Experimental results demonstrate that CBAM yields more significant performance improvements than using CAM or SAM individually.

**Table 3:** Results of ablation experiments with the C-CFF module.

Module	mIoU (%)	Parameters (M)	FLOPs (G)	FPS
CFF	84.06	2.71	14.83	44.01
CFF+CAM	85.19	2.77	15.04	43.36
CFF+SAM	84.71	2.81	15.16	43.27
CFF+CBAM	85.57	2.89	15.326	42.89

#### 3.4.2 Ablation experiments of different modules

To evaluate the impact of the proposed improvements on DeepLabv3+ performance, we conducted ablation experiments using a self-constructed dataset. The baseline model was DeepLabv3+ with an Xception backbone. Four ablation settings were assessed using standard semantic segmentation metrics. **Table 4** shows the experiment’s results, which√ indicate that the specified module was employed.

1. Group 1: The baseline model’s backbone was replaced with the MobileNetV2 architecture, and the standard convolution operations in the encoder–decoder were substituted with depthwise separable dilated convolutions.
2. Group 2: Building on Group 1, the S-ASPP structure was introduced, followed by the CBAM.
3. Group 3: Based on Group 1, the C-CFF module was incorporated into the decoder to fuse features across different scales.
4. Group 4: The C-CFF module was further integrated with Group 2.

**Table 4:** Results of ablation experiments with each module.

MobileNetV2	S-ASPP	C-CFF	mIoU (%)	Parameters (M)	FLOPs (G)	FPS
			82.72	54.714	167.139	18.4
✓			83.94	2.745	13.145	31.75
✓	✓		84.75	2.791	13.612	33.27
✓		✓	85.43	2.847	15.326	35.31
✓	✓	✓	85.57	2.890	15.767	42.89

**Table 4** presents the results of replacing the Xception backbone with MobileNetV2. This modification, combined with the use of depthwise separable dilated convolutions, improved the mIoU by 1.22%, reduced the number of parameters by 51.97M, decreased FLOPs by 153.99G, and increased the inference speed to 31.75 FPS. The integration of the S-ASPP module further improved segmentation performance, increasing the mIoU by an additional 1.49%, with only slight increases of 0.102M in parameters and 2.18GFLOPs. The introduction of the C-CFF structure further refined the model architecture and contributed to enhanced segmentation accuracy. When all three modules were combined, the model achieved an mIoU of 85.57%, representing a 2.85% improvement over the original configuration. The number of parameters was reduced to 2.89M, FLOPs were decreased to approximately one-tenth of the original value, and the inference speed nearly doubled. Each modification contributed to a more lightweight model design while simultaneously enhancing segmentation accuracy.

### 3.5 Testing on the PASCAL VOC 2012

**Figure 12:**
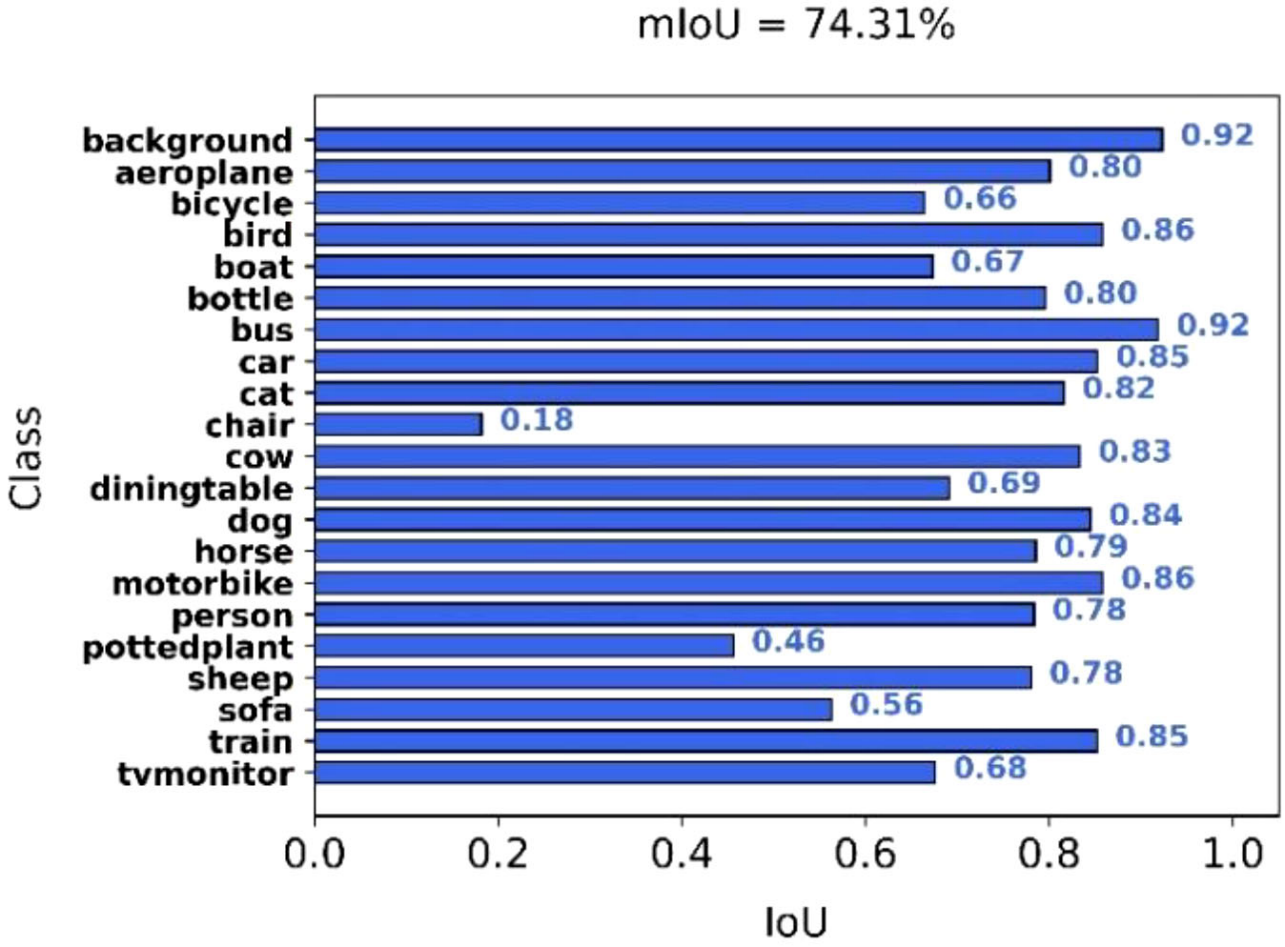
The PASCAL VOC 2012 dataset’s mIoU values for all categories.

The PASCAL VOC 2012 dataset is widely used in computer vision and includes 21 semantic classes, such as car, person, cat, and dog. It serves as a standard benchmark for evaluating semantic segmentation models. We evaluated the performance of the proposed DSC-DeepLabv3+ model on this dataset to assess its generalization capability. As illustrated in **Figure 12**, the model achieves competitive segmentation results. As detailed in **Table 5**, our model achieves higher mIoU compared to several existing methods, including U-Net, FCN, PSPNet, and BiSeNet. Although the mIoU is slightly lower than that of the original DeepLabv3+ with an Xception backbone, our model demonstrates significant advantages in terms of parameter count, computational cost, and inference speed. Specifically, compared with the MobileNetV2-based DeepLabv3+, our model improves mIoU by 1.72%, reduces the number of parameters by 50%, lowers computational cost by 73%, and increases inference speed by 11.88 FPS. Furthermore, compared to BiSeNet, our model achieves 3.69% higher mIoU, reduces parameters by 2.89M, and lowers FLOPs by 34.9G. Compared to ICNet, it achieves 3.42% higher mIoU, with a reduction of 23.61M in parameters and 12.9G in FLOPs. Although the inference speed is 6.05 FPS lower than that of BiSeNet, the proposed model achieves a favorable balance between segmentation accuracy and model efficiency. Overall, the experimental results demonstrate the strong generalization capability of the proposed model.

**Table 5:** Comparison of segmentation results of different models on PASCAL VOC 2012.

Models	Backbone	mIoU (%)	Parameters (M)	FLOPs (G)	FPS
Unet	VGG16	58.78	43.93	184.4	10.3
PSPNet	ResNet50	68.94	46.716	118.47	26.7
DeepLabv3+	Xception	**75.65**	54.714	167.139	16.4
	MobileNetV2	72.59	5.81	56.248	21.68
FCN	VGG16	71.5	32.75	89.8	9.16
BiSeNet	Xception	70.62	5.8	50.3	**39.61**
ICNet	ResNet50	70.89	26.5	28.3	28.5
Ours	MobileNetV2	74.31	**2.89**	**15.326**	33.56

Ablation study results. Bold values indicate the best results.

## 4 Discussion

Semantic segmentation models have found increasing application in agriculture, where large-scale architectures have demonstrated notable performance improvements. However, deploying models with large parameter counts on embedded devices such as agricultural robots and drones remains challenging due to limited computational and memory resources. Although lightweight models reduce parameter counts, they frequently suffer from performance degradation, especially in complex field conditions characterized by high misclassification rates. To overcome these limitations, this study presents DSC-DeepLabv3+, an improved lightweight semantic segmentation model built upon the DeepLabv3+ framework. Specifically, the original backbone is replaced, and standard convolutions in both the ASPP module and decoder are substituted with depthwise separable convolutions, reducing computational complexity. A strip pooling mechanism is incorporated into the ASPP module, forming an S-ASPP structure that enhances the model’s capacity to capture multi-scale contextual information. Furthermore, integrating the CBAM module suppresses background interference and strengthens feature representation. In the decoder, the improved C-CFF module facilitates the efficient integration of multi-stage features, thereby reducing pixel-level information loss and enhancing prediction accuracy. To evaluate the proposed model, a corn–weed segmentation dataset was constructed. Experimental results demonstrate that DSC-DeepLabv3+ achieves an mIoU of 85.57% and an inference speed of 42.89 FPS, with only 2.89M parameters and 15.326 GFLOPs. Although its mIoU is only slightly lower (by 0.71%) than that of the original DeepLabv3+, the proposed model significantly reduces model size and computational overhead. Moreover, it outperforms lightweight baseline models such as BiSeNet under resource-constrained conditions, demonstrating superior efficiency and accuracy. Its generalization capability is further confirmed through evaluation on the PASCAL VOC 2012 dataset.

Despite the encouraging results achieved in this study, several limitations remain. One notable issue is the absence of direct comparisons with recently proposed state-of-the-art lightweight models specifically designed for agricultural scenarios, such as the improved U-Net (Zuo and Li, 2024) and DFFANet (Feng et al., 2022). Although these models are well-recognized in the field and were considered for inclusion, reliable reproduction was impeded due to the lack of publicly available source code and insufficient hyperparameter details in their original publications. Attempts to contact the corresponding authors were unsuccessful. While our experiments included several widely adopted and representative baseline models, the omission of the most recent architectures may limit the completeness of performance evaluation and the positioning of our approach within the current research landscape. In future work, we aim to include such models once reliable implementations become accessible. Another limitation lies in the model’s robustness under complex and variable environmental conditions, such as lighting changes, cluttered backgrounds, and diverse weed morphologies. Although data augmentation was employed to simulate some of these scenarios, real-world agricultural environments are often more unpredictable, featuring strong shadows, overlapping vegetation, and high similarity between foreground and background. These factors may challenge the model’s generalization capability. Furthermore, the current dataset may not adequately capture the full diversity of weed species across different geographic regions and growth stages. To address these issues, future research will focus on expanding the dataset to include more representative field conditions and broader weed categories. Enhancing model robustness through adaptive attention mechanisms, domain generalization techniques, or the integration of multispectral and temporal data will also be explored. Additionally, further optimization of the lightweight architecture will be pursued to support real-time deployment on resource-constrained agricultural platforms, ultimately advancing its applicability in precision agriculture.

## 5 Conclusions

To tackle the challenge of efficient weed identification in maize fields under resource-constrained conditions, this study offers the following key contributions. A maize field weed image dataset was constructed and preprocessed to reflect a wide range of realistic growth conditions. Subsequently, a novel lightweight semantic segmentation model, termed DSC-DeepLabv3+, was proposed. The model maintains a compact architecture, requiring merely 2.89M parameters and 15.236 GFLOPs, thereby addressing memory and processing limitations commonly encountered in field-level deployment. Experimental evaluations show that DSC-DeepLabv3+ achieves an mIoU of 85.57% on the constructed maize weed dataset, surpassing both conventional and lightweight benchmark models. Future work will focus on extending the model to other crop types to enhance its generalizability and contribute to the advancement of precision agriculture.

## Data Availability

The raw data supporting the conclusions of this article will be made available by the authors, without undue reservation.
